# Approaches to participant recruitment and predictors of retention in a large community based public health trial: findings from the building blocks trial

**DOI:** 10.1186/1745-6215-14-S1-O111

**Published:** 2013-11-29

**Authors:** Eleri Owen-Jones, Mike Robling, Julia Sanders, Rebecca Cannings-John, Gwenllian Moody

**Affiliations:** 1 Cardiff University, Cardiff, Wales, UK

## Introduction

Building Blocks is a randomised controlled trial evaluating the effectiveness of the Family Nurse Partnership (FNP) programme in 18 sites in England. FNP is a home visiting intervention aiming to address social exclusion and health disadvantage. Participants were 1600 teenage mothers, living in often challenging social circumstances. Recruitment usually followed an initial approach by a community midwife but also other local health or social care professionals.

## Methods

Tailored recruitment pathways were established following a generic model whereby prospective participants completed a referral slip passed onto local Building Blocks researchers for subsequent approach. Explicit strategies for recruitment and retention were established, the latter in particular was developed in collaboration with the trial's lay stakeholder group. Potential predictors of successful follow-up (telephone and face-to-face interviews up to 24 months following birth) were explored using baseline data (e.g. socio-demographic, psychological, site characteristics).

## Results

Recruitment rates varied considerably by site but with a small time extension exceeded the target. The success of various recruitment strategies will be assessed narratively and with reference to a theory of participation (Social Exchange Theory). Baseline factors found to be predictive of follow-up will be presented. In addition, simple logistical considerations (e.g. contemporaneous verification of contact details by local researchers) played a key role in managing follow-up.

## Discussion

Our recruitment and retention strategies were informed by existing evidence, and were evaluated empirically and against contemporary theory of study participation. Understanding how evidence and theory may need to take into account specific trial circumstances will be explored in this presentation.

